# Commentary: Implications of SARS-Cov-2 infection for pregnancy with diabetes: achievements and open questions for feto-maternal medicine

**DOI:** 10.1186/s12884-021-04046-3

**Published:** 2021-08-21

**Authors:** Christian S. Göbl, Latife Bozkurt, Wolfgang Henrich

**Affiliations:** 1 Department of Obstetrics and Gynaecology, Medical University of Vienna, Waehringer Guertel 18-20, A-1090 Vienna, Austria; 2 Clinic of Obstetrics, Charité-Universitätsmedizin Berlin, Corporate Member of Freie Universität Berlin, Humboldt-Universität zu Berlin, Berlin, Germany; 3 Berlin Institute of Health, Berlin, Germany; 4 Department of Metabolic Disorders and Nephrology, Hietzing Hospital, Vienna, Austria

## Abstract

SARS-Cov-2 (Severe Acute Respiratory Coronavirus 2) infection confers a non-negligible risk for younger pregnant women with diabetes, which is still less well investigated. This topic was recently addressed by a systematic scoping review in *BMC Pregnancy and Childbirth*, aiming to summarize the complex interaction between SARS-Cov-2 infection, pregnancy and diabetes. This commentary will summarize and discuss the main findings of this article and its implications for future research.

## Background

Diabetes mellitus is a major risk factor for a severe course of COVID-19 (Corona Virus Disease 2019) with higher risk for manifesting a viral pneumonia-induced acute respiratory distress syndrome (ARDS) and leading to increased mortality rates [1, 2]. Although, recent systematic reviews found that worse disease severity in diabetic patients is related to higher age and male sex [3], SARS-Cov-2 (Severe Acute Respiratory Coronavirus 2) infection confers a non-negligible risk also for young pregnant women with diabetes, which is still less well investigated or understood. This topic was recently addressed by Eberle et al. who published a systematic scoping review in *BMC Pregnancy and Childbirth*, aiming to summarize the complex interaction between SARS-Cov-2 infection, pregnancy and diabetes. Thereby the authors identified three major topics, with need of further research [4].

## Discussion of the paper

### The pathophysiological implications of SARS-Cov-2 infection for human pregnancy and diabetes

As comprehensively summarized by the authors, the prognosis of COVID-19 is related to several factors including platelet activation and increased proinflammatory cytokine release, which is associated with worsening metabolic control, poor disease progression and consequently poor pregnancy outcomes [4]. Noteworthy, SARS-Cov-2 infection may also negatively affect insulin secretion due to direct effects on β-cell function possibly leading to new onset or exacerbation of the disease [5]. This is particularly important for pregnant patients, as even physiologic pregnancy represents a metabolic stress situation associated with a higher degree of insulin resistance [6]. The described pathophysiologic changes, associated with SARS-Cov-2 infection, have major implications for mothers (e.g. hyperglycemia, hypoxemia, thrombosis or preeclampsia) and potential implications for offspring health as well (e.g. due to transgenerational effects caused by fetal programming and preterm delivery) [4].

### The implications of COVID-19 pandemic for screening and diagnosis of gestational diabetes mellitus (GDM)

It is challenging to uphold GDM screening and diagnosis standards while there is a purpose to limit as possible in-person contact in order to reduce the risk of exposure and dissemination of SARS-Cov-2. Consequently, several health care authorities modified their recommendations for GDM screening and testing during the COVID-19 pandemic. Fasting plasma glucose (FPG), glycated haemoglobin A1c and random plasma glucose were discussed as alternative screening strategies to universal testing by use of oral glucose tolerance tests (OGTT) [4]. In this context it should be mentioned that screening by FPG has the advantage of being cheap and less time consuming [7]. Moreover, data from the HAPO (Hyperglycemia and Adverse Pregnancy Outcome) study showed that FPG is sufficient to identify over 50% of all cases if the IADPSG (International Association of Diabetes in Pregnancy Study Groups) criteria were used for GDM classification [8] and recent studies found that fasting hyperglycaemia is associated with a more severe metabolic phenotype of GDM [9]. The possible advantages of FPG screening (i.e., being cheap, simple and easily to obtain as well as its ability to characterize patients with particularly high risk) may be of major relevance during the actual health care crisis. In addition, FPG can be combined with other risk factors in clinical prediction models, which can further help to reduce the number of more time consuming examinations [10].

### The assessment of optimal treatment strategies and the role of digitalized medicine in the global COVID-19 pandemic

Eberle et al. clearly summarized the potential advantages of “digital care” in the treatment of pregnancies with diabetes [4]. For sure, digitalized medicine will gain more and more importance, especially in the treatment of diabetes. For example, modern real-time continuous glucose monitoring systems (rt-CGMS) enable patients to digitally share their glucose profiles with obstetricians and diabetologists, providing novel opportunities for telemetric interventions. Of note, CGM was recently shown to be effective for pregnant women with type 1 diabetes [11] and research on women with GDM is ongoing [12].

## Conclusion

However, despite our achievements in the pathophysiological understanding of the disease as well as in the development of novel diagnostic strategies and telemetric approaches the effective treatment of diabetic pregnancies during the COVID-19 pandemic is still a great challenge for obstetrics and feto-maternal medicine. Regular appointments with obstetricians are required to assess possible fetal malformations and growth trajectories in fetal ultrasound. Leaving out advanced fetal sonography even at early gestation (e.g. due to limited health care access during the COVID-19 pandemic) can potentially cause severe complications in later pregnancy – for example due malformations which could be otherwise detected already at the first trimester [13]. Research on the complex interaction between SARS-Cov-2 infection, diabetes and pregnancy is still ongoing and it will take years even after the end of the global COVID-19 pandemic to capture all aspects and consequences of the current health care crisis for feto-maternal medicine.

## Data Availability

Not applicable.

## Abbreviations

COVID-19: Corona Virus Disease 2019
ARDS: Acute respiratory distress syndrome
SARS-Cov-2: Severe Acute Respiratory Coronavirus 2
GDM: Gestational diabetes mellitus
FPG: Fasting plasma glucose
OGTT: Oral glucose tolerance test
HAPO: Hyperglycemia and Adverse Pregnancy Outcome
IADPSG: International Association of Diabetes in Pregnancy Study Groups
rt-CGMS: Real-time continuous glucose monitoring systems
